# Incidence of hydatid disease in children: A systematic review and meta-analysis

**DOI:** 10.5339/qmj.2025.115

**Published:** 2025-12-11

**Authors:** Amani N. Alansari, Mohamed Sayed Zaazouee, Marwa Messaoud, Salma Mani, Ksia Amine

**Affiliations:** 1 Department of Pediatric Surgery, Hamad Medical Corporation, Doha, Qatar; 2 Faculty of Medicine, Al-Azhar University, Assiut, Egypt; 3 Pediatric Surgery Department, Fattouma Bourguiba University Hospital, Monastir, Tunisia; 4 Faculty of Medicine, University of Monastir, Monastir, Tunisia *Email: aalansari9@hamad.qa

**Keywords:** Hydatid, echinococcosis, incidence, children, meta-analysis

## Abstract

Hydatid disease (echinococcosis) is a zoonotic infection caused by *Echinococcus* species, characterized by cyst formation in various organs, most commonly the liver and lungs. While often asymptomatic in the early stages, progressive cyst growth can lead to severe complications, including organ dysfunction, secondary infections, or rupture. In this review, we aimed to assess the incidence of hydatid disease among pediatric populations across different regions. We searched PubMed, Scopus, Cochrane, and Web of Science for studies reporting the incidence of hydatid disease in children up to February 20, 2025. A random-effects meta-analysis was performed using STATA version 28. Subgroup analyses were conducted based on country, geographic region, and cyst location. Study quality was assessed using the Newcastle–Ottawa Scale. We pooled data from nine studies, yielding an overall incidence of 3.37 per 1,000 children (95% confidence interval [CI]: 0.52–8.48), with substantial heterogeneity (*I*^2^ = 99.97%). The highest incidence was reported in China (17.86 per 1,000), followed by Turkey (1.48 per 1,000) and Bulgaria (1.47 per 1,000). Lower incidence rates were reported in Iran (0.62 per 1,000) and Romania (0.12 per 1,000). Studies conducted in rural areas showed a higher incidence (14.84 per 1,000) compared to those including patients from diverse geographic regions (1.91 per 1,000). Based on the available evidence, we conclude that the incidence of hydatid cysts in children varies across countries, with the highest rates observed in China and in rural areas. Echinococcosis poses a significant threat to both public health and livestock; therefore, effective monitoring and control strategies are crucial to reduce its impact.

## 1. INTRODUCTION

Hydatid disease (echinococcosis) is a zoonotic infection caused by *Echinococcus granulosus* and *Echinococcus multilocularis*.^1^ Humans become accidental hosts by ingesting food or water contaminated with parasite eggs, leading to cyst formation in various organs.^2^ In approximately 90% of the cases, cysts develop in the liver and lungs, though they can affect almost any organ, particularly in endemic regions. Areas with poor environmental and personal hygiene practices are at higher risk of hydatid disease.^3^ *E. granulosus* causes cystic echinococcosis (CE), characterized by fluid-filled cysts that primarily develop in the liver and lungs. In contrast, *E. multilocularis* causes alveolar echinococcosis (AE), a more aggressive and invasive disease that mimics malignant tumors, primarily affecting the liver and potentially spreading to other organs.^1^ Early-stage infections are often asymptomatic, with clinical manifestations appearing only as the hydatid cysts enlarge. Without proper treatment, the disease can lead to severe complications, with a post-surgical mortality rate of 2.2% and a recurrence rate of 6.5%.^4,5^ It is frequently detected incidentally during routine clinical evaluations or through serologic, radiographic, or ultrasonographic screening.^4^

Hydatid disease is widespread, with an incidence of over 50 new cases per 100,000 in endemic areas and a prevalence reaching 5%–10% in regions such as Argentina, Eastern Africa, Peru, Central Asia, and China.^6^ Globally, it accounts for approximately 184,000 disability-adjusted life years (DALYs) and results in economic losses of approximately $760 million annually.^7^

Although hydatid disease is reported to be less common in children than in adults, this may be due to its silent progression, with symptoms often appearing later in life.^8^ Screening studies have revealed notably high seropositivity rates, ranging from 6% to 13% in children, indicating substantial exposure to *Echinococcus* parasites in endemic regions.^9,10^ When symptomatic, children face a higher risk of complications due to the relatively larger size of cysts in proportion to their body size, which increases the likelihood of cyst rupture and secondary infections.^11^ Quantifying the incidence of hydatid disease in children is crucial, as early identification and intervention can significantly reduce the burden of symptomatic disease later in life. Targeted preventive measures—such as public health education, improved sanitation, and veterinary control programs—can help interrupt transmission cycles, ultimately reducing the incidence and long-term complications.^12^ In this systematic review and meta-analysis, we aim to comprehensively assess and pool the reported incidence of hydatid disease in pediatric populations, providing a clearer understanding of its epidemiological impact and identifying high-risk areas that could benefit from targeted prevention and control strategies.

## 2. METHODS

This systematic review and meta-analysis were conducted in accordance with the guidelines of the Preferred Reporting Items for Systematic Reviews and Meta-Analyses (PRISMA) statement.^13^

### 2.1 Search strategy and study selection

A comprehensive literature search was conducted using Medical Subject Headings (MeSH) and free-text terms across PubMed, the Cochrane Library, Web of Science (WOS), and Scopus, covering publications from inception to February 20, 2025. The following search terms were used: (incidence OR rate) AND (hydatid OR Echinococc) AND (child* OR pediatric* OR paediatric*), to identify relevant studies reporting the incidence of hydatid disease in pediatric populations. Two independent reviewers screened the records to determine their relevance. Studies that appeared relevant based on the title and abstract were subjected to a thorough full-text review. Discrepancies between reviewers were resolved through discussion, with a third reviewer consulted when necessary to reach consensus.

### 2.2 Eligibility criteria

We included studies reporting the incidence of hydatid disease in children under 18 years, based on a combination of clinical, imaging, serological, and pathological findings, rather than relying solely on seropositivity. A diagnosis of CE or AE was considered valid if confirmed through serological testing in conjunction with characteristic imaging findings on computed tomography (CT), magnetic resonance imaging (MRI), chest X-ray, ultrasonography, bronchoscopy, or other radiological modalities. Studies that diagnosed echinococcosis solely on the basis of seropositivity, without imaging or clinical correlation, were excluded. Studies that did not report incidence data, did not specify the population at risk, or did not focus on pediatric cases were also excluded. We included studies published in English, without restrictions on publication date or geographic location.

### 2.3 Data extraction and quality assessment

After reaching consensus on the final selection of eligible studies, two researchers independently extracted relevant data. Key information—including study summaries, baseline characteristics, and outcomes—was systematically recorded in Excel spreadsheets. Key variables collected included study design, country, study period, residence, sample size, age, gender, diagnostic methods, hydatid cyst locations, reported risk factors, geographical distribution, and outcomes. Additionally, outcomes were recorded as the number of events relative to the total population at risk. The quality of the included studies was assessed using the Newcastle–Ottawa Scale (NOS), with two versions applied according to the study design.^14^

### 2.4 Statistical analysis

A random-effects meta-analysis was conducted to pool incidence estimates, appropriately accounting for both within-study and between-study variability. Results are presented as forest plots showing the estimated incidence and corresponding 95% confidence intervals (CIs). Heterogeneity was assessed using Cochran’s Q test and the *I*^2^ statistic.^15^ To explore potential sources of heterogeneity, we conducted subgroup analyses based on the country of study, disease location (hepatic involvement only), and geographic setting (rural vs. mixed/urban populations). When these subgroup analyses did not fully resolve the observed heterogeneity, we performed a leave-one-out sensitivity analysis to assess the influence of individual studies on the pooled incidence estimates. Furthermore, a meta-regression analysis was conducted to evaluate the impact of study-level covariates—including study design (cohort vs. cross-sectional) and data collection period—on the incidence rates.

A funnel plot was generated to visually assess asymmetry and potential publication bias. All statistical analyses were conducted using STATA version 18 (StataCorp, College Station, Texas, USA).

## 3. Results

### 3.1 Search results

**Figure 1:**
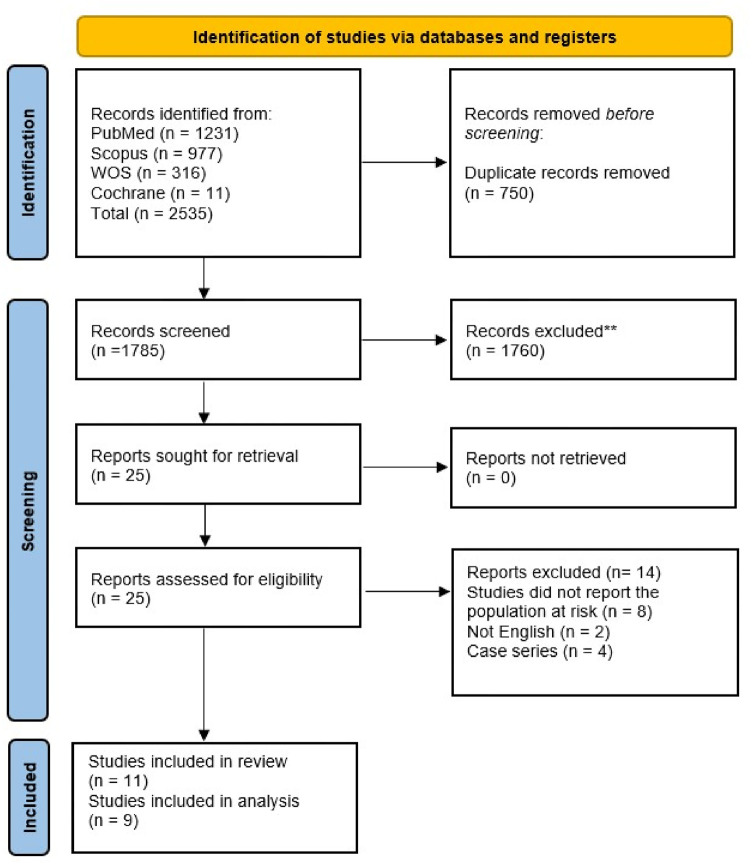
PRISMA flow chart.

Our search identified 2,535 records in total. After removing 750 duplicates, 1,785 studies remained for title and abstract screening. Of these, 25 studies were subjected to a thorough full-text review, and only 11 studies met the inclusion criteria,^9,10,16–24^ with 9 of them included in the meta-analysis.^10,16–22,24^ The PRISMA flowchart is presented in Figure 1.

### 3.2 Study characteristics

The included studies varied in design, spanning both cross-sectional and retrospective cohort methodologies. Studies were conducted across five countries: Iran,^19,20,22^ China,^10,18,24^ Bulgaria,^17^ Romania,^16^ and Turkey.^21^ Sample sizes varied considerably, ranging from a few hundred to over one million participants. The definition of the pediatric population also differed across studies, with most including children and adolescents up to 16–18 years of age, while a few studies extended the upper age limit to 19 years. The included studies covered a wide calendar span, with individual study durations ranging from 1 year to 35 years. The liver and lungs were the most commonly affected organs, with hepatic involvement being dominant in most studies. In contrast, Mamishi et al. reported a higher prevalence of lung involvement compared to hepatic cysts. Diagnostic approaches included imaging techniques such as ultrasound, CT, and MRI, as well as serological tests like ELISA and indirect hemagglutination. The use of pathology for confirmation was emphasized in some studies.^16,19,22^ Geographically, rural populations showed a consistently higher prevalence of CE compared to urban settings, except in one study conducted in Iran.^19^

Key risk factors identified across the included studies were close contact with infected dogs, agricultural exposure, poor hygiene, and home–slaughter practices. The study by Sarkari and colleagues was excluded from the meta-analysis because it relied solely on seropositivity to diagnose hydatid disease, without confirmation through imaging or clinical evaluation.^9^ They reported a seropositivity prevalence rate of 6.7%. Statistical analysis revealed no significant association between seropositivity and age, education level, dog ownership, or residential areas. However, a significant correlation was observed between sex and seropositivity. Another study by Todorov and Boeva was excluded due to overlapping data with a different study by the same author.^23^ A summary of the included studies is presented in Table 1. The quality assessment of the included studies, conducted using the NOS, is presented in Supplementary Tables 1 and 2, with most studies demonstrating good quality.

### 3.3 Meta-analysis

**3.3.1 Overall incidence**

**Figure 2:**
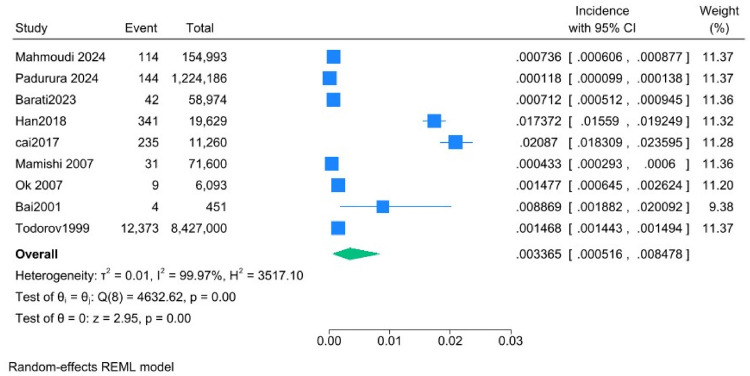
Forest plot of the overall incidence of hydatid disease in children.

**Figure 3:**
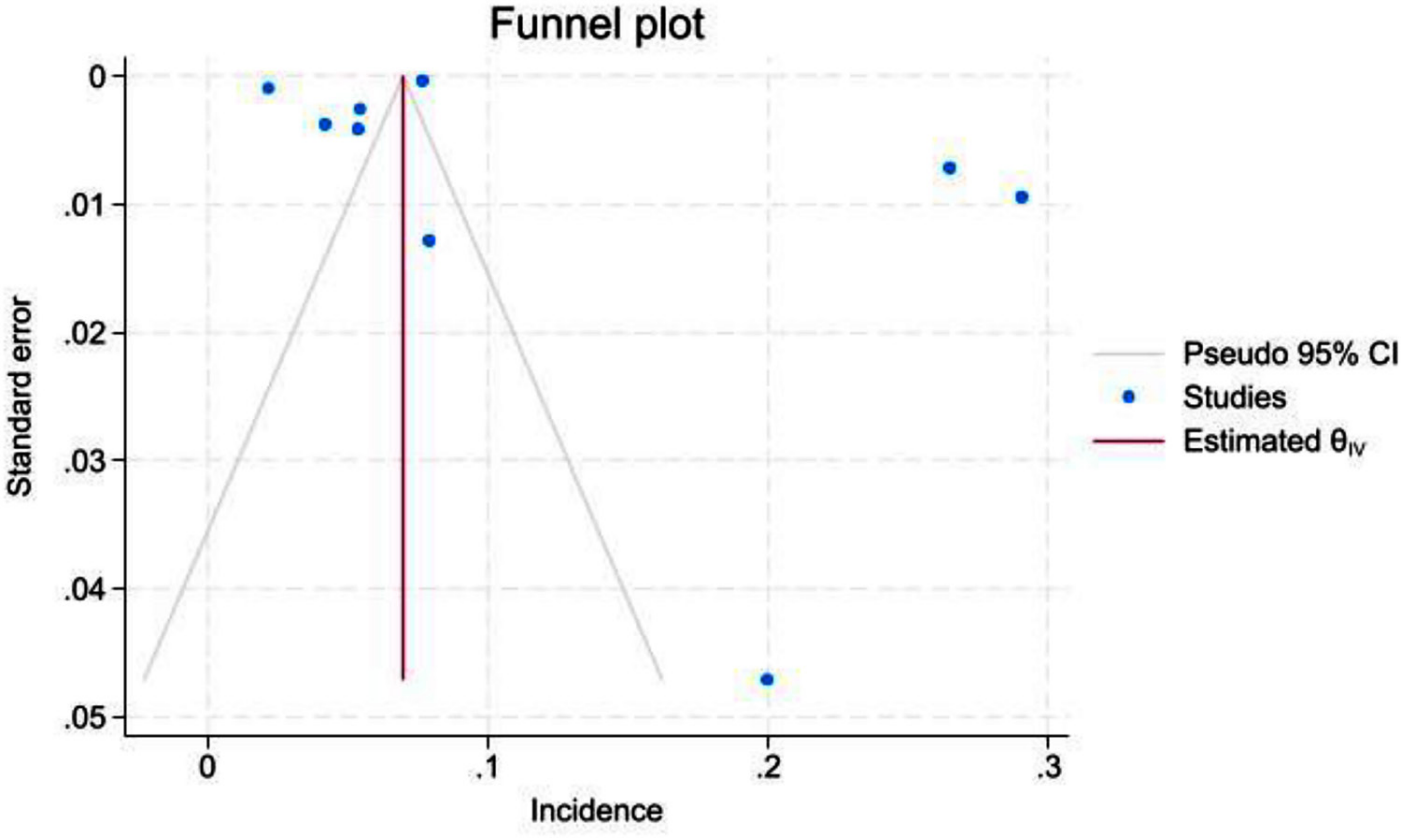
Funnel plot assessing publication bias in studies reporting the incidence of hydatid disease. The observed asymmetry suggests the presence of potential publication bias.

Results of the meta-analysis revealed a pooled incidence of 3.37 per 1,000 (95% CI: 0.52–8.48). Heterogeneity was high (*I*^2^ = 99.97%), indicating substantial variability among the included studies (Figure 2). The funnel plot (Figure 3) showed an asymmetrical distribution of studies, suggesting potential publication bias.

**3.3.2 Incidence across different countries**

**Figure 4:**
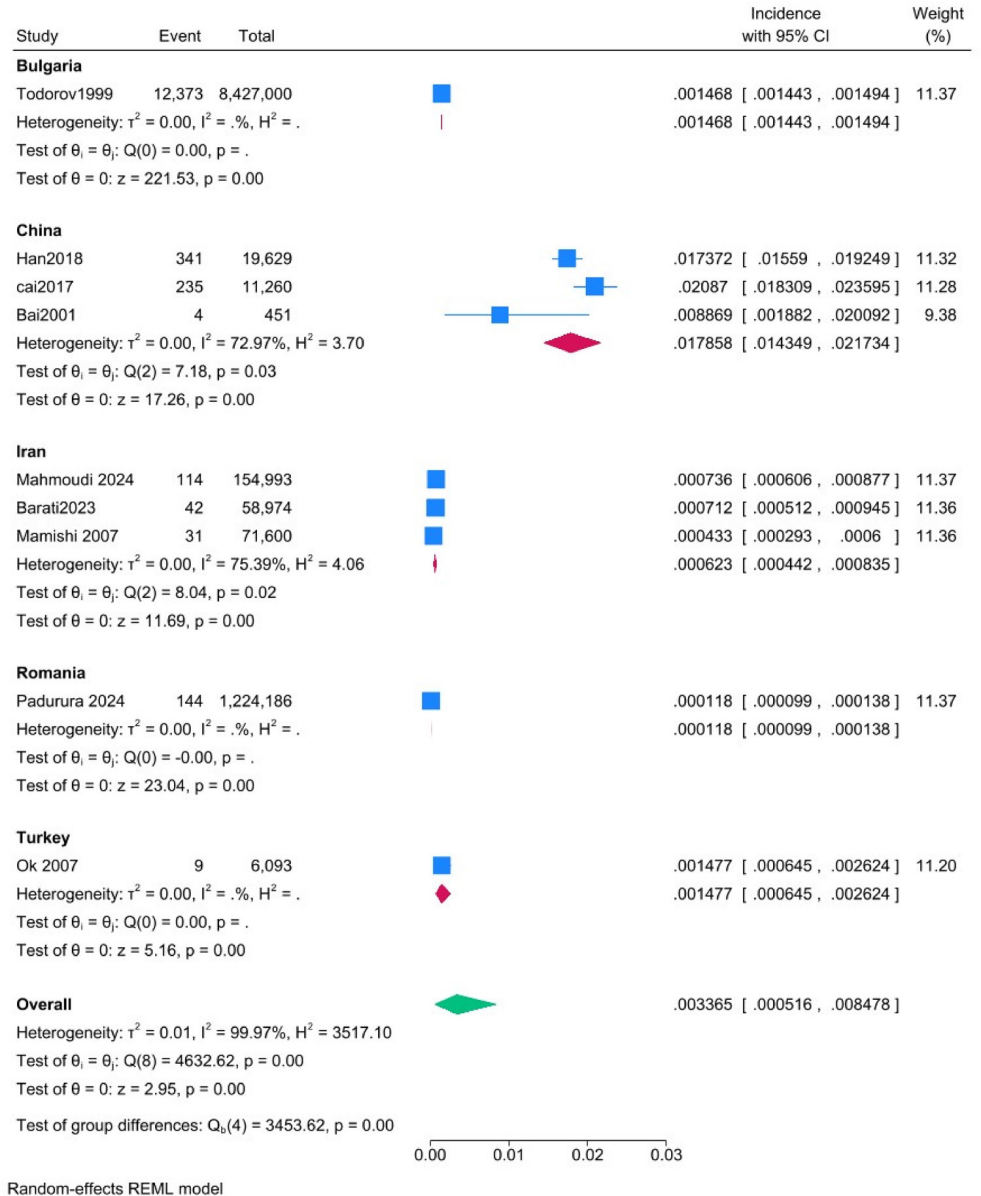
Forest plot of the subgroup analysis comparing pooled incidence rates across different countries.

Figure 4 illustrates the pooled incidence rates across different countries. The highest pooled incidence of hydatid disease was observed in studies from China (17.86 per 1,000; 95% CI: 14.35–21.73), followed by Turkey (1.48 per 1,000; 95% CI: 0.65–2.62), and Bulgaria (1.47 per 1,000; 95% CI: 1.44–1.49). Lower incidence rates were reported in Iran (0.62 per 1,000; 95% CI: 0.44–0.83) and Romania (0.12 per 1,000; 95% CI: 0.10–0.14). Heterogeneity across countries was substantial (*I*^2^ = 99.97%), indicating considerable variability among the included studies. The leave-one-out analysis did not resolve this persistent heterogeneity.

**3.3.3 Incidence based on cyst location**

**Figure 5:**
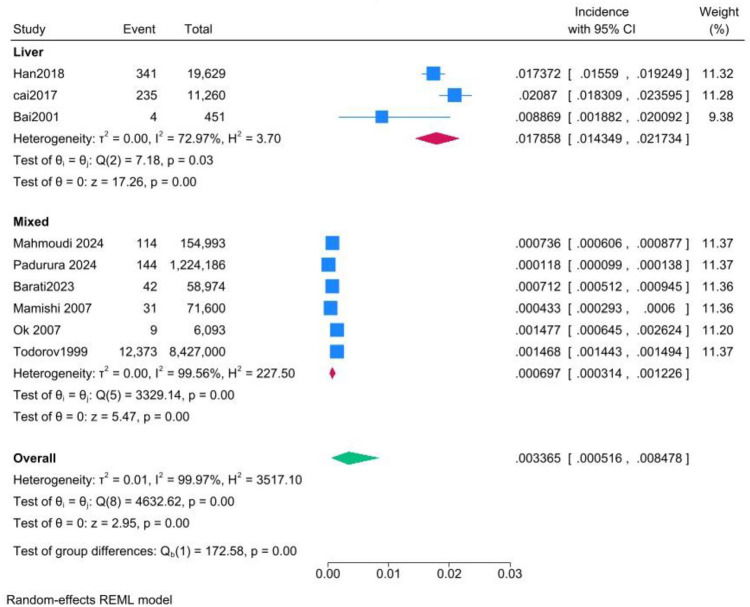
Forest plot of the subgroup analysis comparing pooled incidence rates between studies that focused exclusively on hepatic cysts and those including variable cyst locations.

Another subgroup analysis showed a significantly higher pooled incidence of hydatid disease in studies focusing exclusively on liver involvement (17.86 per 1,000; 95% CI: 14.35–21.73) compared to studies that included cysts at various locations (0.70 per 1,000; 95% CI: 0.31–1.23). Heterogeneity was moderate in the liver-only subgroup (*I*^2^ = 72.97%) but substantially higher in the mixed-location subgroup (*I*^2^ = 99.56%). The difference between subgroups was statistically significant (*p* < 0.01). However, the higher incidence observed in the liver-only subgroup may be influenced by the fact that all three included studies were conducted in China, suggesting potential regional factors rather than true differences based on cyst location (Figure 5). The leave-one-out analysis did not resolve the persistent heterogeneity.

**3.3.4 Incidence based on geographical regions**

**Figure 6:**
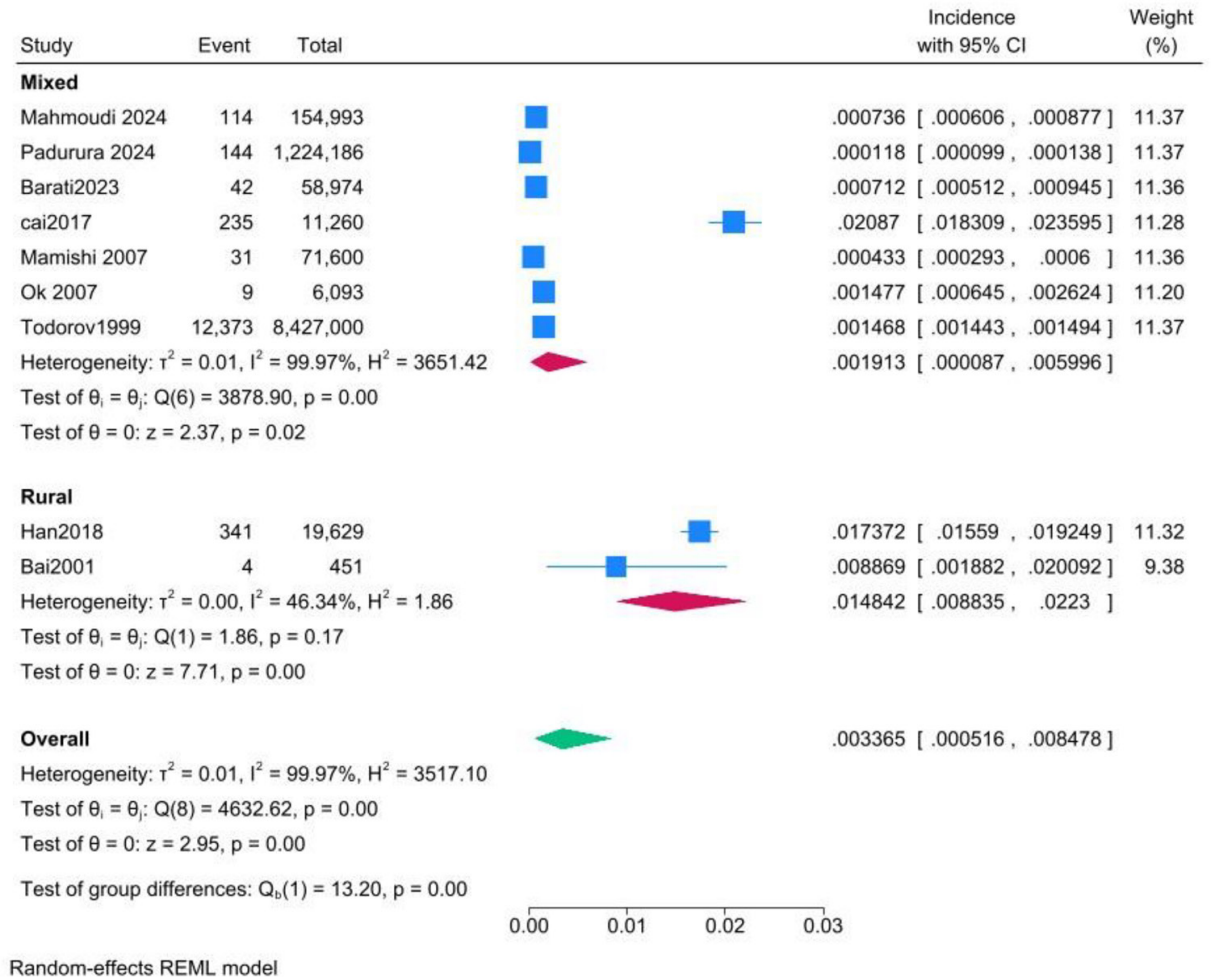
Forest plot of the subgroup analysis comparing pooled incidence rates between studies that focused exclusively on rural regions and those including varied geographical regions.

As shown in Figure 6, the pooled incidence of hydatid disease was significantly higher in studies focusing exclusively on rural regions (14.84 per 1,000; 95% CI: 8.88–22.23) compared to studies that included diverse geographical locations (1.91 per 1,000; 95% CI: 0.87–5.99). Heterogeneity was moderate in the rural-only subgroup (*I*^2^ = 46.34%) but substantially higher in the mixed-location subgroup (*I*^2^ = 99.97%). The difference between subgroups was statistically significant (*p* < 0.01), indicating that geographic factors may influence disease incidence. The leave-one-out test did not resolve the persistent heterogeneity.

Meta-regression analysis was performed to further explore sources of heterogeneity, using study design and data collection period as moderators (Supplementary Table 3). Study design (cohort vs. cross-sectional) was not significantly associated with differences in effect size (*p* = 0.284), indicating that the study design does not account for the heterogeneity observed in incidence estimates reported across studies. In contrast, the data collection period was strongly associated with the effect size. Longer data collection periods (2–35 years) were significantly associated with lower incidence estimates compared to a one-year data collection period (all *p* < 0.001). The negative coefficients indicate an inverse relationship between the length of data collection and the reported incidence. Notably, the meta-regression model accounted for a substantial proportion of the between-study heterogeneity (*I*^2^ = 81.4%), while the residual heterogeneity was low (*I*^2^ < 20%), falling within the range generally considered unimportant (0%–40%).

## 4. DISCUSSION

The meta-analysis estimated a pooled incidence of 3.37 per 1,000 children. Among the included studies, the highest incidence was reported in China (17.86 per 1,000), followed by Turkey (1.48 per 1,000) and Bulgaria (1.47 per 1,000). Lower rates were observed in Iran (0.62 per 1,000) and Romania (0.12 per 1,000). The incidence was significantly higher in rural areas (14.84 per 1,000) compared to mixed-location studies (1.91 per 1,000), indicating a strong geographic influence.

The three studies from China reported the highest incidence rates of hydatid disease. This higher incidence is largely because these studies included both CE and AE, whereas many other studies focused solely on CE.^10,18,24^ China has vast rural and pastoral regions, particularly in western provinces such as Xinjiang, Qinghai, and Tibet, where hydatid disease is endemic due to high livestock density and nomadic farming practices. Many rural communities still rely on traditional farming and herding, which facilitates maintenance of the *Echinococcus* life cycle.^10,18,24^ A previous meta-analysis by Fan et al. reviewed data on bovine hydatid disease in China from 2000 to 2021, analyzing 57 articles and 72 datasets sourced from both Chinese and international databases. The overall prevalence of bovine hydatid disease was estimated at 17.27%. However, infection rates have declined over time, with the lowest prevalence of 7.54% reported since 2016. The highest reported prevalence reached 53.93%,^25^ suggesting that veterinary controls in China remain insufficient. Widespread home slaughtering continues to contribute to an elevated risk of transmission to humans.

The lowest incidence rate in Romania can be attributed to several factors. Veterinary control and slaughterhouse regulations improved significantly after European Union accession, resulting in enhanced animal health monitoring and reduced transmission.^16^ Additionally, stray dog control measures, including sterilization and deworming programs, have reduced the number of infected dogs shedding *Echinococcus* eggs. Finally, the transition from subsistence farming to more industrialized agriculture in Romania has reduced human–animal interactions that facilitate transmission.^16^ Paduraru and colleagues reported that the incidence of CE has decreased over the past 25 years, possibly due to economic changes, aging rural populations, and better disease control measures.^16^

A meta-analysis in Iran estimated the pooled prevalence of hydatid disease at 5%, which is significantly higher than the incidence rate observed among children in our meta-analysis.^26^ This discrepancy may be due to seroprevalence studies detecting past infections or exposure, which can inflate overall prevalence rates compared to studies focusing on active cases in children.

Various risk factors have been reported for hydatid disease, and understanding these differences is crucial for designing targeted prevention and control strategies for specific populations. Most studies reported a higher prevalence of hydatid disease in rural areas.^16,17,20,22^ Interestingly, Mahmoudi and colleagues found a higher prevalence in urban areas in Iran, suggesting differences in transmission dynamics or healthcare access in Tehran compared to rural regions.^19^ Direct contact with infected animals, especially dogs and livestock, has been identified as a major risk factor in multiple studies. Barati et al. reported that 76.2% of cases had a history of animal contact, while Mamishi et al. highlighted the role of exposure to dogs and sheep.^20,22^ Sarkari et al. and Todorov et al. emphasized the presence of stray dogs as a key risk factor, particularly in rural communities.^9,17^ In China, risk factors included a nomadic lifestyle, home slaughter of livestock, and traditional agricultural practices, all of which increase exposure to *Echinococcus* eggs.^10,18,24^ Poor hygiene practices, consumption of contaminated food and water, and inadequate sanitation were identified as risk factors in studies from China^18,24^ and Romania.^16^ Todorov et al. showed that the incidence of hydatid cyst in Bulgaria declined following control measures but resurged when these programs were scaled back.^17,23^

Beyond regional differences, globalization may also influence incidence patterns. Increased international travel, labor migration, and tourism to endemic areas may expose non-endemic populations to *Echinococcus* infection. However, the studies included in this review did not provide data stratified by travel history or mobility, making it difficult to quantify this effect. Future research should specifically investigate whether globalization-related factors contribute to transmission dynamics, particularly in regions not traditionally considered endemic.

Our meta-analysis included incidence data exclusively from Iran, China, Bulgaria, Romania, and Turkey, as no other eligible reports on pediatric incidence were identified. Over the past three decades, the global burden of echinococcosis has remained consistently high, particularly in countries of North Africa and the Middle East.^27^ However, despite the high disease burden in these countries, our review did not identify eligible studies specifically addressing pediatric echinococcosis. This gap may be due to a lack of dedicated research or underreporting within pediatric populations. The absence of pediatric-specific data highlights the need for further epidemiological studies in these regions to better assess the disease burden and improve early diagnosis and management in children. Outside the included countries, other endemic regions have reported substantial pediatric burdens of echinococcosis but lack standardized incidence reporting.^28–31^ These parallels suggest that the observed data gaps may reflect a wider global underestimation of pediatric echinococcosis. Strengthening regional disease surveillance and encouraging the publication of pediatric-specific data are crucial for informing global control strategies.

Early detection is crucial for improving outcomes in pediatric echinococcosis.^32^ Implementing routine screening programs, particularly in endemic areas, can aid in the timely diagnosis and effective management of affected children. Preventive strategies—such as regular deworming programs for dogs, public health campaigns on hygiene and safe animal handling, and improved sanitation—can significantly reduce transmission. Educational initiatives targeting schoolchildren and their families are essential for promoting behavioral changes that minimize exposure to *Echinococcus* eggs.^33^ Our findings highlight the importance of adopting One Health strategies that integrate human and veterinary medicine to effectively interrupt the transmission cycle of echinococcosis.^34^ Real-world successes in countries like New Zealand, Argentina, and Iceland—where the disease has been nearly eliminated—highlight the impact of coordinated efforts, including routine dog deworming, community education, and stringent meat inspection practices.^35,36^ Adapting and implementing similar comprehensive approaches in endemic regions holds promise for significantly alleviating the disease burden, particularly among children who remain one of the most vulnerable yet often overlooked populations. In addition to local residents, preventive measures should also target visitors, including tourists and migrant workers, who may be exposed while staying in endemic regions. Tailored health education and travel advisories can help reduce the risk of transmission among these groups. Ultimately, this review emphasizes that children in endemic regions remain an under-recognized population at risk. Since echinococcosis is largely preventable, these findings should motivate stakeholders to prioritize pediatric screening, implement intersectoral control programs, and invest in surveillance infrastructure in endemic counties.

This meta-analysis provides valuable epidemiological insights into the burden of echinococcosis among children across different populations, highlighting the need for public health interventions. The geographic diversity of the studies enhances the ability to identify the risk factors and disease patterns with greater clarity. Moreover, the generally high methodological quality of the included studies supports the reliability of our findings.

Several limitations should be acknowledged. First, our meta-analysis revealed significant heterogeneity among the included studies, despite efforts to standardize methodology through subgroup analyses by country, disease location, and geographic setting. Sensitivity analysis using a leave-one-out approach did not resolve this heterogeneity, prompting the use of a meta-regression analysis. Meta-regression analysis indicated that study design (cohort vs. cross-sectional) did not significantly explain the variability in effect sizes. However, the data collection period emerged as a significant moderator, with longer data collection intervals associated with lower reported incidence rates. This may reflect an averaging effect over time, changes in diagnostic or reporting practices, or actual epidemiological shifts, such as reduced disease burden. Although this factor accounted for a substantial proportion of the between-study variance, considerable residual heterogeneity remained, indicating the presence of other unmeasured sources of variability. Second, differences in diagnostic methods across studies, particularly in serological tests and imaging techniques, may introduce bias into the results. Third, variations in environmental, socioeconomic, and healthcare access factors among study populations may limit the generalizability of the findings. Fourth, many hospital-based retrospective studies reported only case numbers without providing prevalence rates, leading to their exclusion and potentially omitting valuable data. Fifth, follow-up data on treatment outcomes were rarely reported in the studies analyzed. Apart from Mamishi et al., who documented a 6% recurrence rate following surgery and albendazole therapy, most studies did not provide information on incomplete resolution or recurrence. This limits our ability to conclude long-term disease outcomes in children. Sixth, because we restricted our review to studies published in English to ensure consistency and quality of reporting, there is a potential for language bias. This may have resulted in underrepresentation of incidence data from non-English speaking countries and could partly explain the scarcity of pediatric-specific reports from regions not represented in our included studies. Finally, there is clear evidence of underreporting of incidence rates in several endemic countries—particularly in North Africa and the Middle East, including Egypt, Tunisia, Algeria, Morocco, Libya, Saudi Arabia, and Iraq—which may affect the accuracy of the estimated disease burden.

## 5. CONCLUSION

Based on the available studies, the meta-analysis indicates that the incidence of hydatid disease among children varies significantly across regions, with the highest rates reported in China and much lower rates in Romania and Iran. A higher incidence was observed in rural populations, highlighting the role of environmental and zoonotic factors in disease transmission. Additionally, several endemic countries, especially in North Africa and the Middle East, have limited epidemiological data on pediatric incidence, indicating possible underreporting and gaps in surveillance. Highlighting these groups underscores the need for targeted public health interventions that prioritize veterinary control, community education, and school-based hygiene initiatives in endemic rural areas. Beyond local residents, preventive strategies should also consider travelers and visitors to endemic regions. Finally, future research should aim to enhance surveillance, address regional data gaps, and include non-English literature to provide a more comprehensive assessment of the global pediatric burden.

## COMPETING INTERESTS

The authors have no conflicts of interest to declare.

## DATA AVAILABILITY STATEMENT

The data supporting the findings of this study are available from the corresponding author upon request.

## AUTHORS’ CONTRIBUTION

AA: Conceptualization, data curation, formal analysis, methodology, supervision, writing—original draft, writing—review & editing. MZ: Formal analysis, methodology, writing—original draft, writing—review & editing. MM: Data curation, investigation, writing—review & editing. SA: Data curation, investigation, writing—review & editing. KA: Conceptualization, data curation, writing—review & editing.

## Figures and Tables

**Supplementary Table 1.** Quality assessment of cohort studies using the NOS tool.

Study ID	Type of study	Selection				Comparability	Outcome			Quality
		D1	D2	D3	D4		D5	D6	D7	
Padurura 2024	Cohort		*	*	*	**	*	*	*	Good
Mamishi 2007	Cohort		*	*	*	**	*	*	*	Good
Boeva 2000	Cohort		*	*	*	**	*		*	Good
Todorov 1999	Cohort	*	*	*	*	**	*	*		Good

D1: Is the case definition adequate/representative of the exposed cohort?

D2: Representative of the cases/selection of the non-exposed cohort.

D3: Selection of Controls/ascertainment of exposure.

D4: Definition of Controls/demonstration that outcome of interest was not present at the start of the study.

D5: Ascertainment of exposure/assessment of outcome.

D6: Same method of ascertainment for cases and controls/was follow-up long enough for outcomes to occur?

D7: Non-response rate/adequacy of follow-up of cohorts.

**Supplementary Table 2.** Quality assessment of cross-sectional studies using the NOS tool.

Study ID	Selection				Comparability	Outcome		Quality score
	D1	D2	D3	D4		D5	D6	
Mahmoudi 2024	*	*	*	*	*	*	*	Good
Barati 2023	*	*	*	*		*	*	Good
Sarakari 2019	*	*	*	*	*	*	*	Good
Han 2018	*	*	*	*		*	*	Good
Cai 2017	*	*	*	*	*	*	*	Good
OK 2007	*	*	*	*		*	*	Good
Bai 2001	*	*		*		*	*	Fair

D1: Representativeness of the sample.

D2: Sample size.

D3. Non-respondents.

D4. Ascertainment of the exposure (risk factor).

D5. Assessment of the outcome.

D6. Statistical test.

**Supplementary Table 3.** Meta-regression of effect size by study design and data collection period.

Moderator	Coefficient	Std. Error	z	P-value	95% Confidence interval
**Study Design**					
Cohort	0.0185	0.0173	1.07	0.284	−0.0153, 0.0523
Cross-sectional (ref)	0.0007	0.014	0.05	0.962	−0.0268, 0.0281
**Data Collection Period**					
10 years	−0.0905	0.02	−4.55	<0.001	−0.1295, −0.0515
2 years	−0.0777	0.018	−4.31	<0.001	−0.1131, −0.0424
25 years	−0.0908	0.02	−4.56	<0.001	−0.1298, −0.0518
35 years	−0.0894	0.02	−4.49	<0.001	−0.1284, −0.0504
7 years	−0.091	0.0199	−4.57	<0.001	−0.1292, −0.0527
8 years	−0.0917	0.02	−4.53	<0.001	−0.1292, −0.0512
1 year (ref)	0.0901	0.017	5.34	<0.001	0.0576, 0.1243

**Table 1.** Characteristics of the included studies.

Study ID	Study design	Country	Study period	Residence	Sample size	Age	Gender	Diagnostics tests	Hydatid cyst locations	Risk factors	Geographical distribution	Results
Mahmoudi^19^	Cross-sectional study	Iran	2011–2020	Tehran	154,993	2–16 years	Male: 64%, Female: 36%	Pathology (91.2%), CT scan (78.1%), ultrasonography (76.3%), chest X-ray (64%), ELISA serology (24.6%), brain CT (2.6%), MRI (1.8%)	Liver and lung (66%), multiorgan (39%), spleen (6%), brain (2%), kidney (4%), abdomen (1%)	Animal contact history	The prevalence of CE was higher in urban areas than in rural settings in this study	The study found an increasing prevalence of pediatric CE in Iran, with liver and lung involvement being the most common. Most patients had large cysts (>50 mm), and the majority underwent surgical intervention alongside albendazole treatment
Paduraru (2023)^16^	Retrospective cohort study	Romania	1998–2022 (25 years)	Arad and Timis	1,224,186	3–17 years	Male: 49.3%, Female: 50.7%	Imaging (radiography, ultrasonography, CT) and anatomopathological investigation	Liver: 94 cases (65.3%), lung: 43 cases (29.8%), other (spleen, kidneys, peritoneum, pancreas): 7 cases (4.9%)	Rural residence, contact with infected dogs, agricultural exposure, lack of hygiene practices, and ingestion of contaminated food or water	The incidence was higher among rural populations compared to urban residents	The incidence of CE in hospitalized children decreased over the study period. The liver was the most affected organ (65.3%), followed by the lungs (29.8%). Boys were more likely to have lung cysts, while girls were more likely to have liver cysts. Complications were more common in pulmonary CE
Barati^22^	Cross-sectional study	Iran	2014–2021	Gorgan	58,974	3–17 years	Male: 78.5%, Female: 21.5%	Positive serology: 26.2%, positive pathology: 52.4%	Liver: 60%, lung: 36%, spleen: 2.4%, abdomen: 2.6%	Contact with animals: 76.2%, living in rural areas: 73.9%	The prevalence of hydatidosis was significantly higher in rural areas compared to urban areas	Hydatidosis was more common among village boys aged 3–9 years. Liver and lung involvement were common, necessitating surgical intervention in most cases
Sarkari et al. (2020)^9^	Cross-sectional study	Iran	2017	Kazeroon	578	6.8 years (±3.7)	Male: 298, Female: 280	ELISA	Lungs, liver	Sex (higher in females), presence of stray dogs	The study was conducted in rural communities in Fars Province, Southern Iran	The seroprevalence of hydatid cysts in children was 6.7% (39 out of 578 children). Seropositivity was higher in females (8.9%) than in males (4.7%)
Han et al.^24^	Cross-sectional study	China	2011–2012	Tibetan	19,629	6–18 years	Male: 9,954, Female: 9,675	Ultrasound: 341 students were identified with echinococcosis (119 with CE and 222 with AE. Serological tests: 2,137 students tested positive for echinococcosis antibodies out of 16,969 participants	Liver	Nomadic lifestyle, traditional agricultural work and animal husbandry, poor sanitation and hygiene practices, home slaughter of livestock, roaming stray dogs	The study primarily focused on rural areas, specifically the Qinghai–Tibetan primary school students in Qinghai Province	A total of 341 students (1.7%) had echinococcosis, with 119 cases of CE (0.6%) and 222 cases of AE (1.1%). The highest AE prevalence occurred in Tehetu (12.1%) and Moba (11.8%) townships in Dari County
Cai^18^	Cross-sectional study	China	NR	Qinghai	11,260	6–16 years	Male: 5,650, Female: 5,610	US in 235 children and IgG antibody	Liver	Frequent contact with infected dogs, poor hygiene, and economic conditions	Higher prevalence in rural areas	The prevalence of echinococcosis among schoolchildren in Golog Tibetan Autonomous Prefecture was 2.1% (235/11,260), with 0.8% for CE and 1.3% for AE. The prevalence was higher in girls and increased with age
Mamishi^20^	Retrospective cohort study	Iran	1995–2005	Tehran	71,600	8.3 years	Male: 58%, Female: 42%	Serological tests: 96% of patients were positive, which translates to approximately 30 out of 31 patients CT scans:14 patients, all of whom showed positive results USG positive in 15 patients	Lung: 24 cases (77%) Liver: 15 cases (48%) Simultaneous liver and lung cysts: 8 cases (26%) Multiorgan involvement: 3 cases (10%)	Contact with dogs or sheep, parents as farmers	Higher prevalence of hydatidosis among rural populations compared to urban residents	Hydatidosis is endemic in Iran, with the lung and liver being the most common sites of cysts. Most patients had a history of contact with dogs or sheep, and all underwent surgery and albendazole treatment, with a 6% recurrence rate
Ok^21^	Cross-sectional study	Turkey	NR	Manisa	166,766	7–14 years.	NR	Portable US: all 6,093 children were examined using the US Serology (ELISA and indirect hemagglutination): only the 9 diagnosed cases were tested with serology	Liver (67%), lungs, spleen, and kidneys	No significant relationship was found with dog ownership, livestock ownership, food hygiene, socioeconomic status, or education level	The prevalence of CE is higher in rural areas compared to urban areas	The prevalence of CE among primary schoolchildren was 0.15% (9 cases). No significant relationship was found between risk factors and infection
Bai^10^	Cross-sectional study	China	NR	Tibetan	198,000	8–18 years	F:M=3:1	ELISA: 41 seropositive cases (9.1% of the total sample) IHA: 7 seropositive cases (1.6% of the total sample) Ultrasound: 4 confirmed cases of hepatic CE (0.98% of the total sample)	Liver	Older pupils and females, hunting, the number of sheep owned by a family	All participants were from rural areas	Seropositivity rate for CE was 9.1% (41/451) using ELISA and IHA Ultrasound confirmed hepatic CE in 0.9% (4/451) of the pupils
Todorov and Boeva (2000)^23^	Retrospective cohort study	Bulgaria	1971–1995 (25 years)	NR	NR	0–19 years	Males: 48.1% Females: 51.9%	Surgically confirmed (5,874)	Lungs: 51.8% Liver: 38.3%	Higher incidence in rural populations, particularly among children and adolescents	Higher prevalence in rural areas	Higher incidence in adults: The annual incidence of CE was higher in adults (3.12/100,000) than in children and adolescents (1.48/100,000), but most infections with Echinococcus granulosus occur during childhood and adolescence
Todorov and Boeva^17^	Retrospective cohort study	Bulgaria	1950–1995	NR	8,427,000	0–14 years	NR	1950–1962: 6,469 new surgically confirmed cases 1971–1982: 2,094 new surgically confirmed cases 1983–1995: 3,780 new surgically confirmed cases	Liver, lungs	Close contact with infected dogs, rural residence, slaughterhouse exposure, and poor control measures for dog infestation	The incidence was higher among rural populations compared to urban residents	Echinococcosis incidence decreased significantly after control measures (1971–1982: 2.0/100,000) but resurged after the reduction of control programs (1983–1995: 3.3/100,000). Higher prevalence in rural areas and in older individuals

CE: Cystic echinococcosis; AE: Alveolar echinococcosis; ELISA: Enzyme-linked immunosorbent assay; MRI: Magnetic resonance imaging; CT: Computed tomography; IHA: Indirect hemagglutination assay; NR: Not reported; USG: Ultrasound sonography; US: Ultrasound scanner.
